# Outcomes of Abrupt Switch to Bevacizumab of Patients Undergoing Aflibercept Intravitreal Injections for Neovascular Age-Related Macular Degeneration in a Tertiary Center in Lombardy, Italy: A Real-Life Retrospective Analysis

**DOI:** 10.1155/2021/7940297

**Published:** 2021-09-11

**Authors:** Alessandro Randazzo, Raffaele Raimondi, Giovanni Fossati, Mary Romano, Tania Sorrentino, Carlo Castellani, Costanza Rossi, Giuseppe Cancian, Paolo Vinciguerra

**Affiliations:** ^1^IRCCS Humanitas Research Hospital, Eye Center, Via Manzoni 56, 20089 Rozzano, Italy; ^2^Department of Biomedical Sciences, Humanitas University, Via Rita Levi Montalcini 4, 20090 Pieve Emanuele, Milan, Italy; ^3^Multidisciplinary Department of Medical, Surgical and Dental Sciences, University of Campania Luigi Vanvitelli, Neaples, Italy

## Abstract

**Purpose:**

To assess real-life anatomical and functional outcomes of switch to bevacizumab in patients undergoing aflibercept intravitreal injections for nAMD.

**Methods:**

Retrospective chart review of all patients diagnosed with nAMD and undergoing intravitreal injections of aflibercept who switched to bevacizumab after the resolution XI/1986 of Lombardy Region.

**Results:**

Among 128 patients undergoing intravitreal injections, a total of 29 eyes of 29 patients met all inclusion criteria and were included in the statistical analysis. Best corrected visual acuity and central macular thickness did not change significantly (*p* > 0.05) between baseline, after the loading phase, and at the last follow-up.

**Conclusion:**

Switching to bevacizumab has been safe and efficacious in patients responding to the loading phase. According to our results, the restrictions imposed by Lombardy Region did not cause any harm to patients undergoing intravitreal anti-VEGF injections.

## 1. Introduction

### 1.1. Background

Age-related macular degeneration (AMD) is a leading cause of progressive loss of vision. The introduction of intravitreal injections (IVI) of vascular endothelial growth factor inhibitors (anti-VEGF) has revolutionized the prognosis of neovascular age-related macular degeneration (nAMD), and they are currently the gold standard of treatment [1]. Among all anti-VEGF agents, ranibizumab, bevacizumab, and aflibercept are the most used in clinical practice.

On 23^rd^ July 2019, a new resolution (XI/1986) about the pharmacological management of nAMD was approved by Lombardy Region (Italy). On the basis of the scientific results of noninferiority of bevacizumab IVI compared to ranibizumab and aflibercept [2], the regional representatives established a reimbursement of 55.60 euros for single bevacizumab administration. Lombard hospitals were thus addressed to use preferably bevacizumab in patients served by the National Health Italian System. On the 8^th^ of August 2020, the regional administrative court (TAR) canceled the latter resolution (N. 01533/2020 REG.PROV.COLL.), reintroducing the possibility of prescribing any type of anti-VEGF as the first line therapy.

Two major phase III clinical trials (VIEW1 and VIEW2) [3] have been carried out to demonstrate noninferiority of aflibercept compared to ranibizumab for treating nAMD. The first study led to aflibercept approval by the FDA in the United States, and the second led to its approval in other Western and Asian countries. Their results showed that over 95% of patients receiving aflibercept injections lost less than three lines at 52 weeks compared to over 94% of patients receiving ranibizumab [3], and bevacizumab has been used as an off-label treatment for nAMD since 2005. Four major prospective trials were carried out to evaluate the efficacy of this drug: the CATT [4], the IVAN [5], the GEFAL [6], and the LUCAS [7] studies. All these studies demonstrated that the visual acuity gain in the bevacizumab group was not significantly inferior to the ranibizumab group.

According to published literature, bevacizumab is not inferior to aflibercept, and the switch has been safe and effective in a small cohort of patients [2, 8]. However, since a similar abrupt change has never been reported previously, the aim of the study was to assess real-life outcomes of switch to bevacizumab in patients undergoing aflibercept intravitreal injections for nAMD.

## 2. Methods

This is a retrospective, observational case study performed in a single center: Istituto di Ricovero *e* Cura a Carattere Scientifico (IRCCS) Humanitas Research Hospital, Rozzano, Milan. The study was conducted in accordance with the principles of the Declaration of Helsinki. The study was approved by the Ethical Committee of Humanitas Research Hospital Rozzano (protocol n.20/21) and all patients signed consent for the retrospective chart analysis.

### 2.1. Patients

A retrospective chart review of all patients diagnosed with nAMD and undergoing intravitreal injections of aflibercept who switched to bevacizumab after the resolution XI/1986 of Lombardy Region was carried out.

Inclusion criteria were a minimum follow-up period of 6 months and a minimum of 4 intravitreal injections of bevacizumab.

We define “switch” as the data of the last aflibercept injection, “loading phase” as the data 3 months after intravitreal injections of bevacizumab, and “last follow up” as the data of the last available follow-up (>6 months).

### 2.2. Outcome Measures

The primary outcomes were the change in best corrected visual acuity (BCVA) and in central macular thickness (CMT).

### 2.3. Clinical Assessment Protocol

All patients underwent a comprehensive ophthalmic examination at each follow-up (f/u) visit. The ophthalmic examination protocol included BCVA assessment in Snellen fractions, applanation tonometry, slit lamp biomicroscopy, dilated fundus examination and spectral domain- (SD-) OCT imaging. The diagnosis of nAMD was confirmed by fluorescein angiography.

### 2.4. SD-OCT Scan Protocol

We used a Spectralis SD-OCT (Heidelberg Engineering GmbH, Heidelberg, Germany). At each visit, the following scans were acquired in all eyes: a high-definition horizontal fovea-centered cross line B-scan at 30° and a horizontal macula raster consisting of 49 B-scans 120 *µ*m spaced over an area of 20°.The ‘‘Thickness Map” function was used to measure automatically the mean CMT, based on the mean retinal thickness within a circular area of 0.5 mm radius from the foveal center.

Two masked observers (R.R. and G.F.) independently evaluated OCT images quantitatively and qualitatively. A third observer (A.R.) resolved any case of disagreement. Segmentation errors were manually corrected with built-in software when needed.

### 2.5. Treatment Protocol

All patients received IVI in our center after signing informed consent. IVI of anti-VEGF was carried out in the operating room by skilled surgeons or trainees under supervision. Before the switch, a treat and extend protocol was performed for injections of aflibercept. After the switch to bevacizumab, patients underwent a loading phase of 3 monthly injections of bevacizumab followed by a treat and extend protocol with bevacizumab injections.

### 2.6. Safety Assessment

At each f/u visit, we investigated frequency and severity of any adverse event.

### 2.7. Statistical Analysis

To carry out the statistical analysis, we used STATA/IC 16 software and converted all Snellen BCVA values into logarithm of the minimum angle of resolution (logMAR) units. All data were expressed as mean–standard deviation. For the comparison of continuous variables, we performed the Wilcoxon signed-rank test. For the comparison of categorical variables, we used McNemar's chi-squared test. The differences were considered statistically significant if *p* value was <0.05.

## 3. Results

Among 128 patients undergoing intravitreal injections, a total of 29 eyes of 29 patients met all inclusion and were included in this observational analysis. The median age of all included patients was 81,4 (±7,9) years, and 13 patients were female and 16 were male. Baseline characteristics are given in Table 1.

All included patients previously received at least three injections of aflibercept.

On average, all 29 eyes had received a mean of 4.45 (±2.55) intravitreal injections of bevacizumab. The final follow-up visit was performed 17.7 ± 10.2 weeks after initiation of bevacizumab treatment.

**Figure 1:**
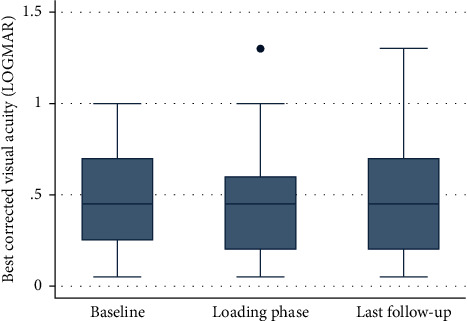
Boxplot analysis illustrates best corrected visual acuity in the eyes at the analyzed timepoints. The ordinate shows best corrected visual acuity (logMAR) at baseline prior to treatment switch (left side), after the loading phase which is three injections of bevacizumab (middle), and at last available follow-up visit (right side) shown on the abscissa. Statistically significant results (pairwise comparison Wilcoxon test, *p* < 0.05) are marked with an asterisk.

Mean BCVA at switch was logMAR 0.508 (±0.054), mean BCVA after the loading phase was 0.504 (±0.064), and mean BCVA at last follow-up was 0.524 (±0.070). No statistical difference was found neither between BCVA at switch and BCVA after the loading phase (*p*=0.74) nor between BCVA at switch and BCVA at last follow-up (*p*=0.76). (Figure 1).

**Figure 2:**
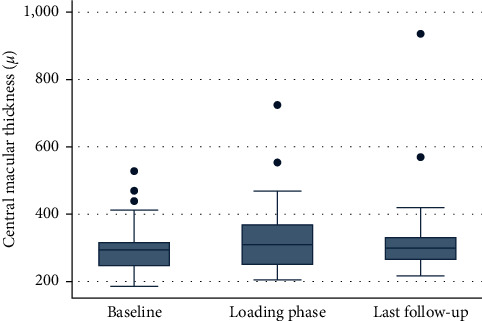
Boxplot analysis illustrates central macular thickness in *μ*m at the analyzed timepoints. The ordinate shows central macular thickness in *μ*m for the eyes at baseline prior to treatment switch (left side), after the loading phase which is three injections of bevacizumab (middle), and at last available follow-up visit (right side) shown on the abscissa. Statistically significant results (pairwise comparison Wilcoxon test, *p* < 0.05) are marked with an asterisk.

Mean CMT at switch was 304 *µ*m (±15), mean CMT after the loading phase was 329 *µ*m (±21), and mean CMT at last follow-up was 336 *µ*m (±26). No statistical difference was found neither between CMT at switch and CMT after the loading phase (*p*=0.29), neither between CMT at switch and CMT at last follow-up (*p*=0.11). (Figure 2).

No severe complications have been reported, and mild complications include 3 cases of conjunctival hyperemia which resolved with topical antibiotics.

## 4. Discussion

Although several studies describe the switch from bevacizumab or ranibizumab to aflibercept, the reverse switch is poorly described in literature.

Because of the abrupt change in nAMD management occurred in Lombardy (Italy) from 23rd July 2019, Lombard ophthalmologists have been led to use preferably bevacizumab. To our knowledge, a similar forceful switch has never been reported previously, and our study aims to assess the outcomes of this IVI shift.

Our results show noninferiority of bevacizumab against aflibercept after a loading phase of three injections and at the final follow-up in terms of anatomical and functional outcomes. Indeed, we found no statistically significant differences in terms of visual acuity and CMT during follow-up. This is in agreement with Waizel et al. [8], who reported an equivalent anatomical effect in nAMD eyes treated with a switching from aflibercept to bevacizumab or the reverse.

IVI of anti-VEGF agents have been shown to be highly effective in treating nAMD and have considerably reduced the burden of visual impairment worldwide [1]. However, therapeutic regimens require periodic IVI which may result in tachyphylaxis. The latter, defined as a progressive reduction of the therapeutic response after repetitive administration of a pharmacologically active substance, has been reported and thoroughly described in several studies [9–12].

Since the current standard of care for neovascular AMD is based on monotherapy [1, 9, 13], switching from one anti-VEGF agent to another represents a valuable option to increase the therapeutic response [10, 14, 15]. According to our results, in case of tachyphylaxis, a switch from aflibercept to bevacizumab may be a viable option.

A good safety profile with no severe complications has been observed in 116 overall IVI of our study. Reported complications include 3 cases of conjunctival hyperemia which resolved with topical antibiotics. We have retrospectively analyzed a sample from real-life clinical routine in which a switch from aflibercept to bevacizumab has been performed without a clinical indication. Our results indicate that the treatment with bevacizumab after previous aflibercept regimen can lead to functional and anatomical preservation, without any significant adverse effects.

We identified a small number of patients (*n* = 6) that did not respond to bevacizumab after three injections and were switched back to aflibercept. No response is intended as no improvement in BCVA and CMT after three bevacizumab IVI. Further studies are necessary to identify common characteristics of “non-responders.”

Our real-life experience highlights that switching to bevacizumab is safe and effective even if some patients may not respond and therefore need to be switched back to aflibercept.

Our study has some limitations: first, the limited sample and the retrospective single center design limit the strength of these results; moreover, the follow-up period and the number of injections are not homogeneous in the analyzed population. Finally, due to the retrospective design of the study, our sample is composed by patients who could have underwent different anti-VEGF treatments in other centers before starting with aflibercept; thus, it was not possible to retrieve the detailed history for the statistical analysis.

To conclude, this study shows that switching to bevacizumab has been safe and efficacious in patients responding to the loading phase. According to our results, the restrictions imposed by Lombardy Region did not cause any harm to patients undergoing IVI.

We highlight how bevacizumab can be a viable option in case of reduced funds or insurance restrictions and can be used as a first line therapy in nAMD with good anatomical and functional results and with a good safety profile even in patients previously treated with aflibercept.

## Figures and Tables

**Table 1:** Baseline demographics and clinical findings.

	*N* = 29
Sex, male, *n*(%)	16 (65%)
Age (standard deviation)	80 (75–87)
Right eye, *n*(%)	14 (48%)
Phakic, *n*(%)	9 (31%)
BCVA, logMAR mean (standard deviation)	0.508 (0.054)
Central macular thickness, *µ*m mean (standard deviation)	304 (15)

## Data Availability

The data used to support the findings of this study are available from the corresponding author upon request.
